# MRI diagnosis of concurrent perforated appendicitis and acute calculous cholecystitis in late pregnancy: a case report

**DOI:** 10.3389/bjbs.2026.16647

**Published:** 2026-10-26

**Authors:** Quang Huy Huynh, Minh Kha Tong Thi, Xuan Quynh Nguyen, Thu Hang Ho Thi, Chi Phong Nguyen

**Affiliations:** 1 Radiology Department, Binh Dan Hospital, Ho Chi Minh City, Vietnam; 2 Radiology Department, Pham Ngoc Thach University of Medicine, Ho Chi Minh City, Vietnam; 3 Radiology Department, University of Health Sciences-Viet Nam National University, Ho Chi Minh City, Vietnam; 4 Vinh Long Government Health Department, Vinh Long, Vietnam; 5 Obstetrics and Gynecology Department, Vinh Long General Hospital, Vinh Long, Vietnam

**Keywords:** appendicitis, cholecystitis, magnetic resonance imaging, peritonitis, pregnancy

## Abstract

**Background:**

Acute appendicitis in pregnancy often presents atypically, and diagnostic delay increases the risk of perforation. Concomitant biliary pathology can further obscure the clinical picture, particularly in late gestation.

**Case:**

A 19-year-old woman at 35 weeks’ gestation presented with 1 day of dull right lower quadrant pain with associated right upper quadrant discomfort. Laboratory testing showed leukocytosis (WBC 16.34 K/μL; neutrophils 82.8%) and a markedly elevated hs-CRP (239.21 mg/L). Ultrasound demonstrated an impacted gallbladder neck stone, but the appendix was not visualized. MRI identified an enlarged appendix (14 mm) with periappendiceal inflammation and moderate free fluid consistent with perforated appendicitis with peritonitis, and concurrently demonstrated imaging features of acute calculous cholecystitis with mild bile duct dilatation. Emergency laparoscopy confirmed a necrotic, leaking appendix with localized purulent peritonitis, and laparoscopic appendectomy was performed. Recovery was uncomplicated, and the pregnancy progressed to an uncomplicated vaginal delivery at 38 weeks’ gestation of a healthy newborn.

**Conclusion:**

In late pregnancy, overlapping right-sided abdominal pain may reflect concurrent surgical pathology. MRI can rapidly clarify diagnosis and guide timely operative management to reduce maternal-fetal risk.

## Introduction

Acute appendicitis is the most common non-obstetric surgical emergency in pregnancy, yet diagnosis remains challenging because pregnancy-related physiological and anatomical changes can mask typical features and shift the appendix away from its usual position [1, 2]. As gestational age increases, the enlarging uterus may displace the cecum and appendix superiorly and laterally, altering the location of maximal tenderness and reducing the reliability of classic peritoneal signs [3]. In addition, nausea, vomiting, mild leukocytosis, and nonspecific abdominal discomfort are common during pregnancy, further reducing the diagnostic specificity of symptoms and basic laboratory tests. Diagnostic delay is clinically important: complicated appendicitis (gangrene, perforation, peritonitis) is associated with substantially worse maternal and fetal outcomes compared with uncomplicated disease [4, 5].

Imaging therefore plays a central role in balancing two competing risks: missing or delaying treatment of true appendicitis versus exposing mother and fetus to unnecessary surgery (negative appendectomy) or ionizing radiation. Large observational data have demonstrated that negative appendectomy in pregnancy is not benign; fetal loss and adverse obstetric outcomes can occur even when the appendix is normal, highlighting the need for improved diagnostic accuracy before operative intervention when feasible [1, 2]. Current imaging strategies generally recommend graded compression ultrasound as the first-line test, with MRI as the next step when ultrasound is nondiagnostic, because MRI provides high diagnostic performance without ionizing radiation [6].

The differential diagnosis of right-sided abdominal pain in late gestation is broad and includes obstetric causes, gastrointestinal disease, urologic conditions, and hepatobiliary pathology. Biliary disease is also common during pregnancy because hormonal changes promote gallstone formation and biliary stasis; acute cholecystitis can present with right upper quadrant pain and systemic inflammation and may coexist with other surgical pathology [7]. When sonography identifies biliary disease, there is also a risk of anchoring on a single diagnosis and overlooking a concurrent intra-abdominal process. Guidelines support laparoscopic approaches for common surgical problems in pregnancy with appropriate pregnancy-adapted technique and multidisciplinary care [7, 8].

We report a 35-week pregnant patient with overlapping right lower quadrant and right upper quadrant pain, marked inflammatory biomarkers, and inconclusive ultrasound, in whom abdominal MRI established the diagnosis of perforated appendicitis with peritonitis and concomitant acute calculous cholecystitis. This case emphasizes the diagnostic pitfalls of right-sided abdominal pain in late pregnancy and the value of MRI in clarifying concurrent surgical pathology without radiation.

## Case presentation

A 19-year-old woman at 35 weeks’ gestation presented in late gestation with a 1-day history of dull right lower quadrant pain associated with right upper quadrant discomfort. She reported no fever. On examination, the abdomen was soft with localized tenderness in the right iliac fossa; there was no guarding or rebound tenderness.

Laboratory tests showed leukocytosis (WBC 16.34 × 109/L, neutrophils 82.8%) and markedly elevated inflammation (hs-CRP 239.21 mg/L).

Ultrasound demonstrated a mildly distended gallbladder (transverse diameter 39 mm) with an impacted gallbladder neck stone (∼16 mm) without obvious wall thickening. Right iliac fossa fat infiltration was also noted, while the appendix was poorly visualized; fetal assessment showed a viable fetus (Figure 1).

**FIGURE 1:**
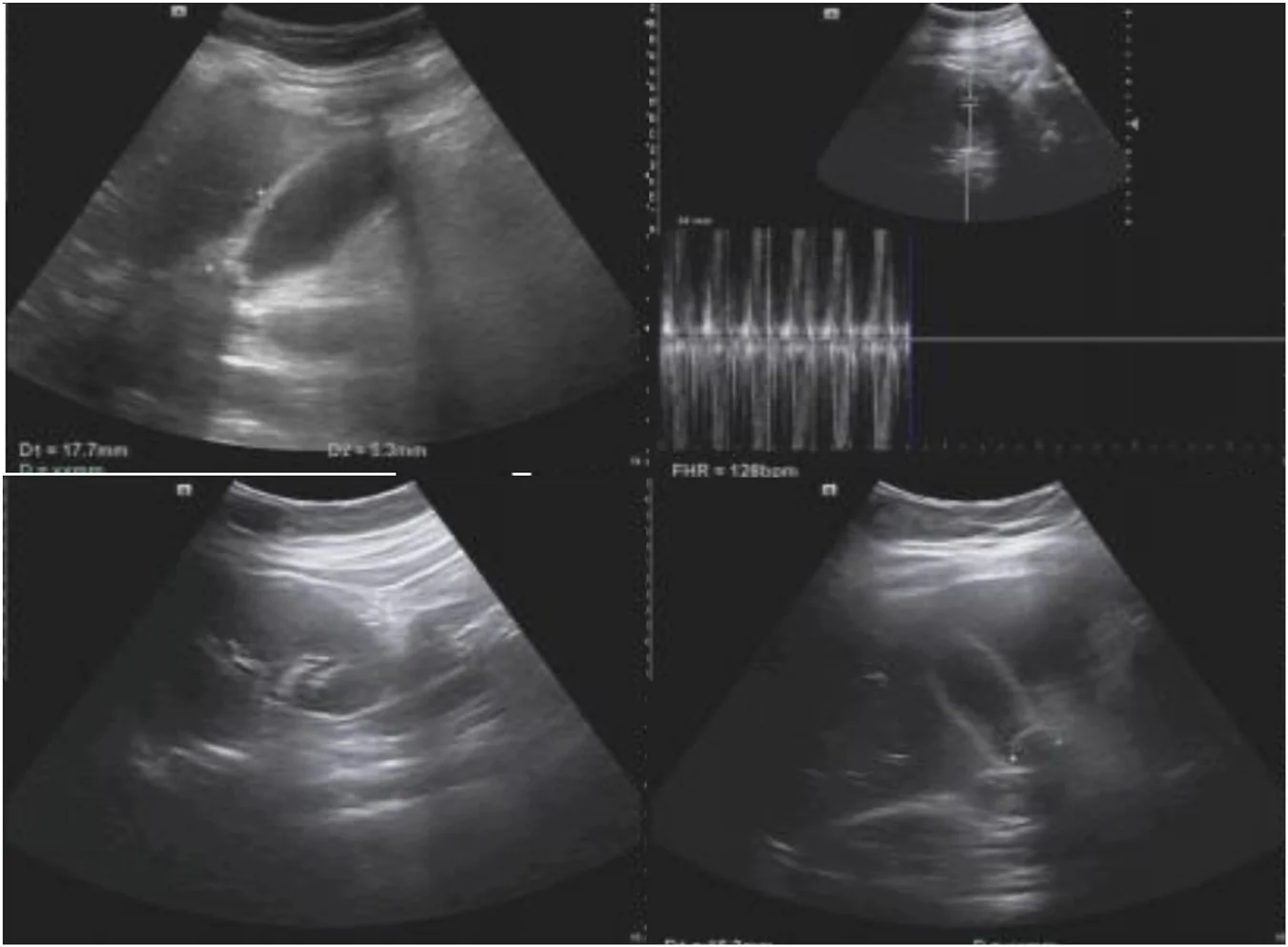
Initial ultrasound showing an impacted gallbladder neck stone and right iliac fossa fat infiltration; the appendix was not confidently visualized.

Given persistent concern for an intra-abdominal surgical process and a nondiagnostic appendix evaluation on ultrasound, abdominal MRI was performed in late gestation. The diagnostic images available for this report comprised axial and coronal T2-weighted sequences; no gadolinium-enhanced images are presented or relied upon for the diagnostic findings described here. MRI identified a markedly enlarged tubular structure consistent with the appendix near the right adnexa (∼14 mm) with luminal fluid and surrounding periappendiceal inflammatory change. The inflamed appendix was further confirmed on paired planes (axial and coronal T2-weighted images), supporting acute appendicitis with significant periappendiceal inflammation (Figures 2A,B).

**FIGURE 2:**
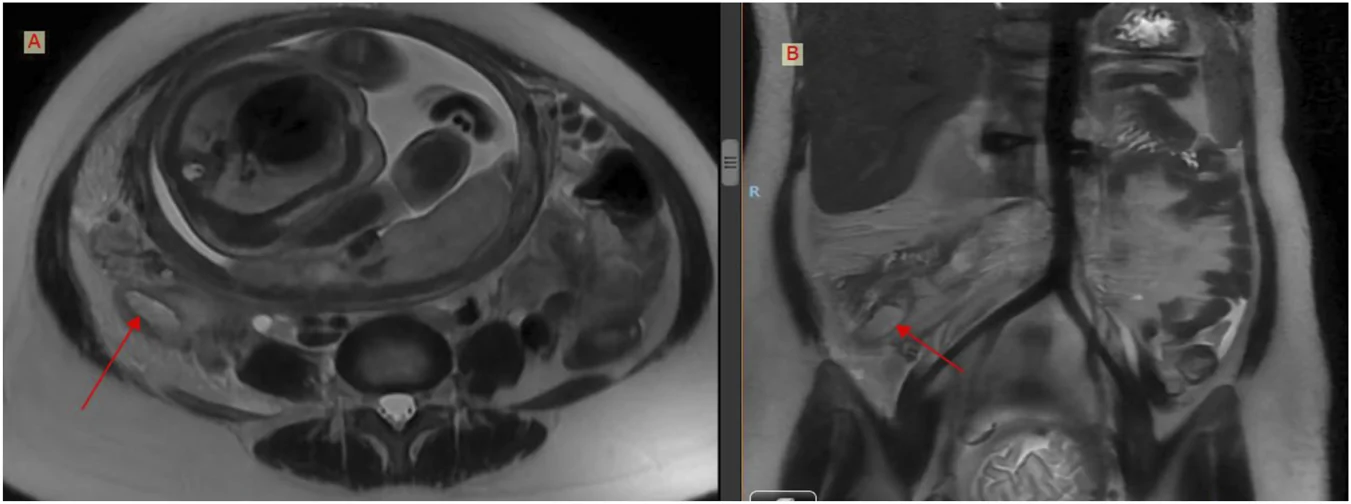
Appendix on MRI in two planes: **(A)** axial T2W and **(B)** coronal T2W showing appendiceal dilation with surrounding inflammatory change and small adjacent fluid (arrows).

MRI also demonstrated hepatobiliary abnormalities: a mildly distended gallbladder (∼82 × 29 mm) with an impacted neck stone (∼15 mm), mild gallbladder wall thickening/edema (∼4.5 mm), and pericholecystic inflammatory change, consistent with acute calculous cholecystitis (Figures 3A,B). The combination of an impacted neck stone, wall thickening/edema, and pericholecystic inflammatory change favored primary acute calculous cholecystitis rather than isolated reactive gallbladder edema; however, because perforated appendicitis with localized peritonitis was present concurrently, a secondary reactive inflammatory component could not be completely excluded. In addition, free intraperitoneal fluid (predominantly in both iliac fossae, right > left) and inflammatory fat stranding centered in the right lower quadrant/right iliac fossa (periappendiceal/paracecal region) were present, raising concern for complicated appendicitis with localized peritonitis (Figures 4A,B). Mild common bile duct (CBD) dilatation (6.2 mm) was noted without MRI evidence of choledocholithiasis; the obstructing calculus was located at the gallbladder neck, compatible with cystic duct obstruction. Available liver biochemistry did not suggest biliary obstruction. Mild bilateral hydronephrosis (grade I) was also noted.

**FIGURE 3:**
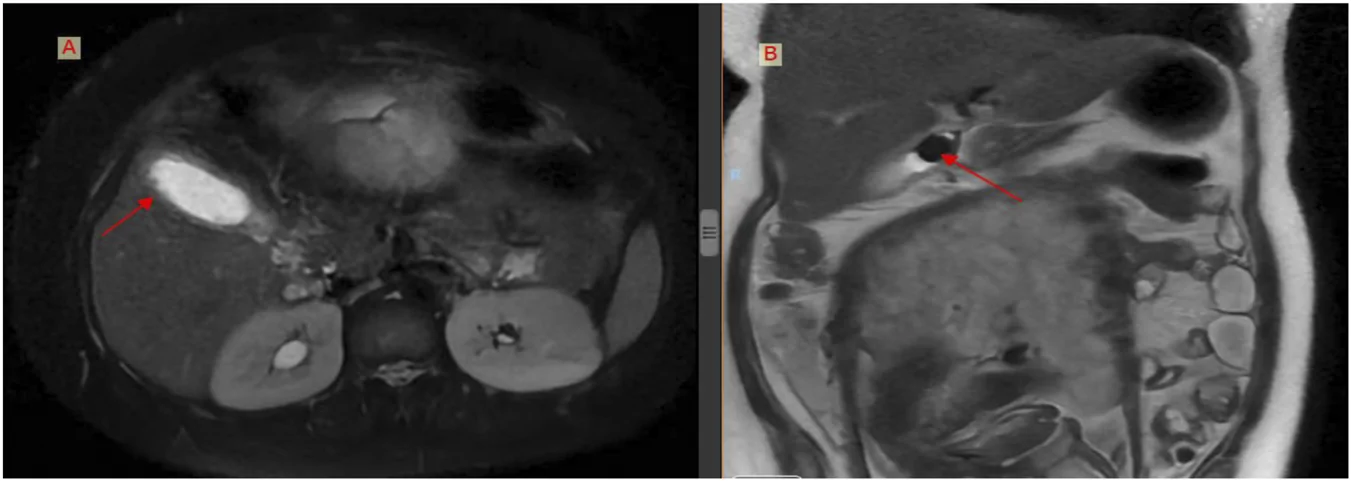
MRI features of acute calculous cholecystitis: **(A)** axial T2W showing gallbladder wall thickening/edema and pericholecystic inflammatory change; **(B)** coronal T2W demonstrating an impacted stone at the gallbladder neck (arrows).

**FIGURE 4:**
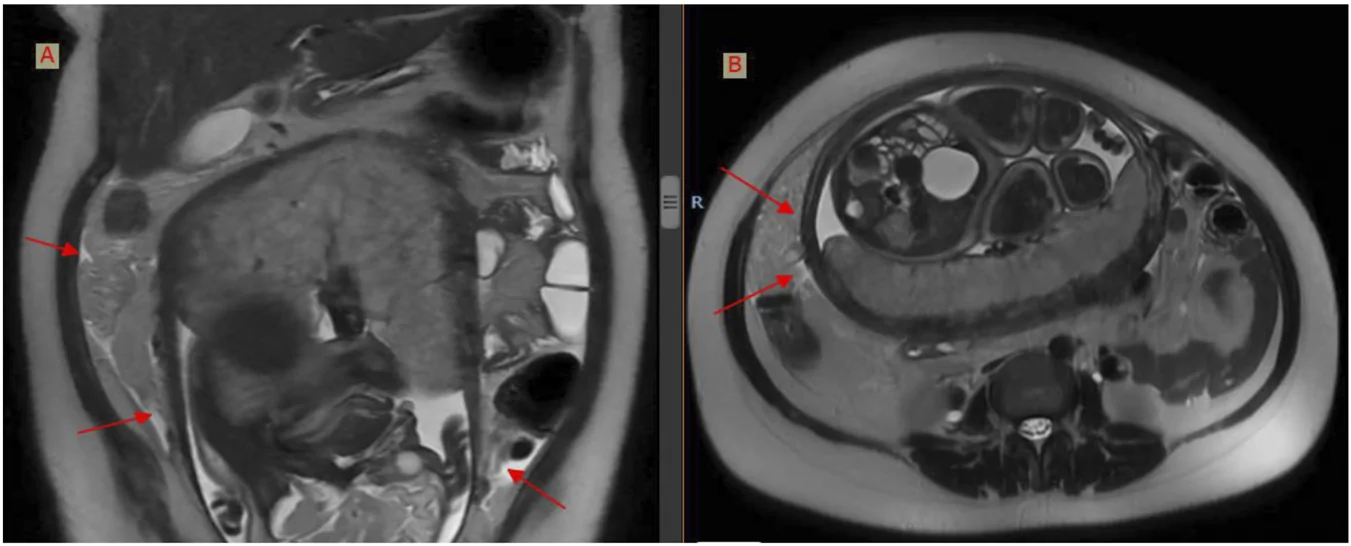
Peritoneal involvement: **(A)** coronal T2W showing free intraperitoneal fluid along both paracolic gutters and iliac fossae (arrows); **(B)** inflammatory fat stranding in the right lower abdomen (arrow).

Emergency laparoscopy was performed the following day after multidisciplinary surgical and obstetric evaluation. Entry and port placement were adjusted for the enlarged uterus and established under direct vision using a pregnancy-adapted technique. Intraoperatively, the appendix was identified in the right iliac fossa and appeared gangrenous with distal perforation, dense omental adhesions, fibrinous exudates, and localized purulent fluid in the right hemiabdomen, consistent with localized peritonitis. Laparoscopic appendectomy was completed successfully. Because the perforated gangrenous appendix with localized purulent peritonitis represented the immediate source requiring urgent control, appendectomy was prioritized. The gallbladder appeared inflamed but was not tense, and in the absence of MRI evidence of choledocholithiasis or biochemical evidence of biliary obstruction, no gallbladder-directed procedure was performed during the index operation. Histopathological examination confirmed complicated acute appendicitis with transmural inflammation, necrosis, and periappendicitis. These histopathological and operative findings were concordant with the MRI evidence of an enlarged inflamed appendix, marked periappendiceal inflammatory change, and peritoneal fluid.

Postoperatively, the patient received antibiotic therapy and underwent routine clinical monitoring. Obstetric ultrasonography confirmed ongoing fetal viability with reassuring parameters. Recovery was uneventful, and the patient was discharged in stable condition. Subsequent follow-up ultrasonography showed resolution of the gallbladder inflammatory changes, and no additional gallbladder-directed treatment was required.

The pregnancy subsequently progressed to term, culminating in an uncomplicated vaginal delivery at 38 weeks of gestation. The newborn was healthy, with a birthweight of 3200 g and Apgar scores of 8 at 1 min and 9 at 5 min.

The patient expressed relief after her postoperative recovery and was reassured by the continued wellbeing of her pregnancy. She was satisfied with the clinical care received and was particularly pleased that the pregnancy progressed to an uncomplicated vaginal delivery of a healthy infant.

A chronological summary of the clinical course is provided in Table 1.

**TABLE 1:** Timeline of the clinical course.

Timepoint	Key event
Initial presentation (35 weeks’ gestation)	One day of right lower quadrant pain with right upper quadrant discomfort; leukocytosis and markedly elevated hs-CRP
Initial imaging	Ultrasound showed an impacted gallbladder neck stone and right iliac fossa fat infiltration; the appendix was not confidently visualized
After nondiagnostic ultrasound	Abdominal MRI demonstrated an enlarged inflamed appendix with periappendiceal inflammation and free fluid, together with gallbladder neck stone, wall edema, and pericholecystic inflammation
Following day	Emergency laparoscopy confirmed gangrenous perforated appendicitis with localized purulent peritonitis; laparoscopic appendectomy was performed, while the gallbladder was managed conservatively
Postoperative follow-up	Recovery was uneventful; follow-up ultrasonography showed resolution of gallbladder inflammatory changes without additional gallbladder-directed treatment
38 weeks’ gestation	Uncomplicated vaginal delivery of a healthy 3200-g newborn (Apgar scores 8 and 9 at 1 and 5 min, respectively)

## Discussion

This late-gestation case illustrates a high-risk diagnostic scenario: right-sided abdominal pain with severe inflammation, nondiagnostic ultrasound for appendicitis, and simultaneous evidence of gallbladder pathology. Three learning points deserve emphasis: diagnostic strategy, interpretation of MRI when more than one pathology is present, and operative prioritization in the third trimester.

Diagnosis is difficult in late pregnancy because anatomical displacement and reduced abdominal wall compliance can blunt guarding and rebound, while physiological leukocytosis can obscure interpretation of inflammatory markers. Delayed diagnosis increases the probability of perforation and peritonitis, which are linked to worse obstetric outcomes [9]. Conversely, negative appendectomy is not benign; fetal loss and adverse obstetric events have been reported even when pathology is absent [10]. These competing harms explain why structured imaging pathways are increasingly recommended.

Ultrasound is widely used as first-line imaging, but non-visualization of the appendix is common as gestation advances due to uterine enlargement, bowel gas, and limited acoustic windows in the right lower quadrant [3]. Evidence-based strategies support escalation to MRI when ultrasound is nondiagnostic [1, 11]. MRI has high diagnostic accuracy for appendicitis in pregnant patients; a pregnancy-specific meta-analysis reported sensitivity around 91.8% and specificity around 97.9% [11, 12], supporting MRI as a reliable tool to reduce both missed appendicitis and unnecessary surgery.

MRI was particularly valuable here because it simultaneously evaluated the appendix, peritoneal fluid, and biliary system without radiation. Typical MRI findings supportive of appendicitis include appendiceal dilation, wall thickening, periappendiceal inflammatory change, and free fluid; signs suggesting perforation include substantial peritoneal fluid, phlegmon, or abscess [13, 14]. For acute cholecystitis, MRI can show gallbladder distension, wall thickening/edema, pericholecystic inflammatory change, and an obstructing calculus. In this patient, the impacted gallbladder neck stone together with wall edema and pericholecystic inflammation favored a concurrent primary biliary process; nevertheless, reactive gallbladder inflammation related to perforated appendicitis and peritonitis cannot be excluded with absolute certainty. By establishing perforated appendicitis with peritonitis while also characterizing the gallbladder abnormalities, MRI helped identify appendiceal source control as the immediate operative priority.

Nonobstetric surgery during pregnancy should not be delayed when clinically indicated, and obstetric consultation and fetal considerations (including appropriate monitoring when feasible) are recommended [15]. Laparoscopy is widely performed in pregnancy with pregnancy-adapted technique: trocar placement adjusted to fundal height, avoidance of aortocaval compression with left lateral tilt, and physiologic monitoring. Society of American Gastrointestinal and Endoscopic Surgeons (SAGES) guidance supports CO_2_ insufflation pressures generally in the 10–15 mmHg range, adjusted to maternal physiology, and emphasizes that access technique (open, Veress, optical trocar) is acceptable in experienced hands when modified for uterine size [2]. In third-trimester laparoscopy, port placement should be individualized according to uterine size, target anatomy, and available working space rather than treated as a single standard configuration. In our case, appendiceal perforation with peritonitis justified emergency operation, and appendectomy provided definitive source control, which was the key determinant of maternal stabilization and fetal wellbeing. Notably, the biliary inflammatory changes resolved on follow-up ultrasonography without the need for cholecystectomy or further gallbladder-directed therapy, supporting a conservative approach when symptoms abate and no ongoing obstruction is suspected. Importantly, maternal recovery was uncomplicated and the pregnancy progressed to a successful term vaginal delivery with a healthy neonate, underscoring that timely source control and multidisciplinary monitoring can result in favorable maternal-fetal outcomes even in late gestation.

A frequent management question in dual-pathology cases is whether to address the gallbladder surgically during the same procedure. Recent SAGES guidelines suggest laparoscopic cholecystectomy rather than nonoperative treatment for acute cholecystitis in pregnancy (conditional recommendation), while in biliary colic during the third trimester either approach may be considered depending on clinical context and resources [2]. Systematic reviews suggest laparoscopic cholecystectomy during pregnancy can be safe in experienced centers, but optimal timing remains individualized and depends on severity, recurrence risk, and gestational constraints [16, 17]. In our patient, the perforated appendix with localized purulent peritonitis was the dominant urgent pathology, whereas the gallbladder was inflamed but not tense and there was no evidence of choledocholithiasis or biochemical biliary obstruction. These findings supported limiting the index operation to appendiceal source control, followed by clinical and ultrasonographic surveillance of the gallbladder. The subsequent resolution of gallbladder inflammatory changes without further intervention supports the individualized conservative decision in this patient, but should not be interpreted as evidence that acute cholecystitis in pregnancy generally requires nonoperative treatment. Clear documentation of biliary findings, postoperative symptom surveillance, and postpartum definitive management if symptoms recur or persistent biliary disease is demonstrated remain pragmatic components of care.

In practice, this case illustrates a pragmatic diagnostic approach for late-gestation right-sided abdominal pain: initial obstetric assessment, first-line ultrasound, and a low threshold for MRI when ultrasound is nondiagnostic or when one imaging abnormality does not fully explain the clinical presentation. MRI can reduce diagnostic anchoring and enable targeted surgical planning, but the sequence described here should be regarded as a clinical lesson from a single case rather than as an established diagnostic algorithm.

The principal strength of this report is the concordance among clinical assessment, ultrasound, MRI, operative findings, histopathology, and maternal-fetal outcome, demonstrating how cross-sectional imaging can clarify competing right-sided abdominal diagnoses in late pregnancy. As a single case report, however, it cannot establish comparative effectiveness, causality, or a general management pathway. In addition, the MRI documentation available for publication is limited to selected T2-weighted images rather than a complete reproducible acquisition protocol, and some gallbladder wall edema or surrounding inflammatory change may have been reactive to the concomitant perforated appendicitis and peritonitis. These limitations warrant cautious interpretation and support framing the report as an illustrative clinical lesson rather than a standardized strategy.

## Conclusion

Concurrent appendicitis and gallbladder disease should be considered in pregnant patients with mixed right lower quadrant and right upper quadrant pain. In late gestation, when ultrasound does not fully explain the presentation, MRI can identify concurrent abnormalities and help prioritize timely management without fetal ionizing-radiation exposure; the management decisions in this report remain specific to the individual clinical context.

## Data Availability

The raw data supporting the conclusions of this article will be made available by the authors, without undue reservation.
